# Interleukin-6 as surrogate marker for imaging-based hypoxia dynamics in patients with head-and-neck cancers undergoing definitive chemoradiation—results from a prospective pilot trial

**DOI:** 10.1007/s00259-021-05602-x

**Published:** 2021-11-13

**Authors:** Alexander Rühle, Nicole Wiedenmann, Jamina T. Fennell, Michael Mix, Juri Ruf, Raluca Stoian, Andreas R. Thomsen, Peter Vaupel, Dimos Baltas, Anca-L. Grosu, Nils H. Nicolay

**Affiliations:** 1 Department of Radiation Oncology, Medical Center – University of Freiburg, Faculty of Medicine, University of Freiburg, Robert-Koch-Str. 3, 79106 Freiburg, Germany; 2 German Cancer Consortium (DKTK), Partner Site Freiburg and German Cancer Research Center (DKFZ), Heidelberg, Germany; 3 Department of Nuclear Medicine, Medical Center – University of Freiburg, Faculty of Medicine, University of Freiburg, Freiburg, Germany

**Keywords:** Head-and-neck cancer, Radiotherapy, Interleukin-6, Hypoxia, FMISO-PET, Biomarker

## Abstract

**Purpose:**

Intratumoral hypoxia increases resistance of head-and-neck squamous cell carcinoma (HNSCC) to radiotherapy. [^18^F]FMISO PET imaging enables noninvasive hypoxia monitoring, though requiring complex logistical efforts. We investigated the role of plasma interleukin-6 (IL-6) as potential surrogate parameter for intratumoral hypoxia in HNSCC using [^18^F]FMISO PET/CT as reference.

**Methods:**

Within a prospective trial, serial blood samples of 27 HNSCC patients undergoing definitive chemoradiation were collected to analyze plasma IL-6 levels. Intratumoral hypoxia was assessed in treatment weeks 0, 2, and 5 using [^18^F]FMISO PET/CT imaging. The association between PET-based hypoxia and IL-6 was examined using Pearson’s correlation and multiple regression analyses, and the diagnostic power of IL-6 for tumor hypoxia response prediction was determined with receiver-operating characteristic analyses.

**Results:**

Mean IL-6 concentrations were 15.1, 19.6, and 31.0 pg/mL at baseline, week 2 and week 5, respectively. Smoking (*p*=0.050) and reduced performance status (*p*=0.011) resulted in higher IL-6 levels, whereas tumor (*p*=0.427) and nodal stages (*p*=0.334), tumor localization (*p*=0.439), and HPV status (*p*=0.294) had no influence. IL-6 levels strongly correlated with the intratumoral hypoxic subvolume during treatment (baseline: *r*=0.775, *p*<0.001; week 2: *r*=0.553, *p*=0.007; week 5: *r*=0.734, *p*<0.001). IL-6 levels in week 2 were higher in patients with absent early tumor hypoxia response (*p*=0.016) and predicted early hypoxia response (AUC=0.822, *p*=0.031). Increased IL-6 levels at week 5 resulted in a trend towards reduced progression-free survival (*p*=0.078) and overall survival (*p*=0.013).

**Conclusion:**

Plasma IL-6 is a promising surrogate marker for tumor hypoxia dynamics in HNSCC patients and may facilitate hypoxia-directed personalized radiotherapy concepts.

**Trial registration:**

The prospective trial was registered in the German Clinical Trial Register (DRKS00003830). Registered 20 August 2015

**Supplementary Information:**

The online version contains supplementary material available at 10.1007/s00259-021-05602-x.

## Introduction

With 5-year survival rates between 40% and 70% depending on tumor stage and localization, the outcome of locally advanced head-and-neck squamous cell carcinoma (HNSCC) is capable of improvement [1]. Surgery with adjuvant (chemo)radiation or definitive chemoradiation are the main treatment modalities for patients with locally advanced HNSCCs of the oropharynx, hypopharynx, and larynx [2–5]. Despite recent developments in surgery and radiotherapy technique as well as improvements in supportive treatments, the outcomes of human papillomavirus (HPV)-negative HNSCC patients only moderately improved over the last decades [1, 6].

It is known for several decades that tumoral hypoxia negatively impacts the efficacy of radiotherapy and therefore worsens the prognosis of HNSCC patients [7–10]. There have been made various attempts to target tumor hypoxia using, e.g., administration of carbogen plus nicotinamide or nitroimidazole [11, 12]. Furthermore, selective dose escalation to hypoxic subregions within the gross tumor volume (GTV) is another strategy that has been studied [13–15]. On the other side, tumor hypoxia dynamics may be used as decision tool to select HPV-positive patients that are suitable for treatment de-escalation [16, 17]. Although there is level 1a evidence in favor of adding hypoxic modification to (chemo)radiation, hypoxic modification is not routinely administered in the clinics [18]. In this context, identification of patients that exhibit a higher degree of tumor hypoxia at baseline or during treatment and may therefore benefit most from hypoxic modification is an important step. Although fluorine F-18 misonidazole positron emission tomography ([^18^F]FMISO PET) is a reliable and valid noninvasive imaging method for tumor hypoxia [19–21], it is demanding for the treatment center and for the patients, especially if [^18^F]FMISO PET/CT is performed in radiotherapy treatment position with a thermoplastic head immobilization mask.

Interleukin-6 (IL-6) is a pleiotropic pro-inflammatory cytokine and has been shown to serve as a negative prognostic parameter for several tumor entities including HNSCC [22, 23]. IL-6 plays an important role in immune regulation, inflammation, and oncogenesis and increases the radioresistance, immune evasion, and metastatic potential of HNSCC [24, 25]. Recent studies have also shown that hypoxia leads to an upregulation of IL-6, providing a rationale to investigate the association between plasma IL-6 concentration and tumor hypoxia dynamics as quantified by [^18^F]FMISO PET [26–28]. Additionally, it has been demonstrated that IL-6 trans-signaling upregulates osteopontin levels, a well-known endogenous plasma hypoxia marker, and *vice versa*, osteopontin was found to increase IL-6 levels at least in chondrocytes [29–31].

In this analysis of a prospective imaging trial, we aimed to explore the role of IL-6 plasma levels during chemoradiation as a potential surrogate for PET imaging-based tumor hypoxia dynamics in HNSCC patients.

## Methods

### Patient treatment

The prospective pilot trial was registered in the German Clinical Trial Register (DRKS00003830) and was approved by the Independent Ethics Committee of the University of Freiburg (reference no. 479/12). It was conducted in accordance with the Declaration of Helsinki (revised version of 2008), and written informed consent was obtained from all patients prior to enrolment. Inclusion and exclusion criteria have been reported in the German Clinical Trials Register. In short, patients with histologically confirmed UICC stage III/IV squamous cell carcinoma of the oral cavity, the oro-/hypopharynx, or the larynx could be enrolled in this trial. Further inclusion criteria comprise an ECOG performance status of 0–1, an age above 18 years, adequate renal clearance, and liver function tests as well as adequate blood cell counts in order to allow for concomitant chemotherapy.

Within the trial, 27 patients provided sequential blood samples in weeks 0, 2, and 5 during chemoradiation for IL-6 quantification. Of these 27 patients, 15 patients were treated as part of the initial trial, and 12 patients based on a trial amendment [32]. Median age of our cohort was 61 years (range 41 to 76 years), and most patients were male (*n*=24, 88.9%) (Table 1). The median body mass index (BMI) ranged at 23.5 kg/m^2^ (range 15.2 to 34.5 kg/m^2^). With 17 patients (63.0%) having an Eastern Cooperative Oncology Group (ECOG) Performance Status of 0, performance status was relatively good in our cohort. The relative majority of patients were ex-smokers (*n*=12, 44.4%), and only 6 patients (22.2%) had no history of previous tobacco consumption. More than half of patients suffered from oropharyngeal cancers (*n*=16, 59.3%) followed by hypopharyngeal and multilevel carcinomas (both *n*=4, 14.8%). HPV-positive oropharyngeal cancers were diagnosed in 10 patients (37.0%).

**Table 1:** Patient and tumor characteristics of the trial cohort with available IL-6 levels (*n*=27). BMI, body mass index; ECOG, Eastern Cooperative Oncology Group; HPV, human papillomavirus; IL-6, interleukin-6

Age (years)		
Median	61	
Minimum	41	
Maximum	76	
BMI (kg/m^2^)		
Median	23.5	
Minimum	15.2	
Maximum	34.5	
Gender		
Male	24	88.9
Female	3	11.1
ECOG performance status		
0	17	63.0
1	10	37.0
Smoking status		
Never smoker	6	22.2
Ex-smoker	12	44.4
Current smoker	9	33.3
Tumor localization		
Oral cavity	2	7.4
Oropharynx	16	59.3
Hypopharynx	4	14.8
Larynx	1	3.7
Multilevel	4	14.8
T stage		
T1	1	3.7
T2	4	14.8
T3	8	29.6
T4	14	51.9
N stage		
N0	3	11.1
N1	3	11.1
N2	20	74.1
N3	1	3.7
HPV		
HPV-positive	10	37.0
HPV-negative	17	63.0

Patients who were included before 2018 (*n*=15) received definitive chemoradiation with intensity-modulated radiotherapy to a total dose of 70 Gy in 35 fractions to the high-risk planning target volume (PTV) and 50 Gy in 25 fractions to the low-risk PTV. Since 2018, patients (*n*=12) underwent definitive chemoradiation with a simultaneous integrated boost to the macroscopic tumor areas, and the high-risk, intermediate-risk, and low-risk PTVs were treated to doses of 69.3 Gy, 62.7 Gy, and 56.1 Gy in 33 fractions, respectively. Three cycles of cisplatin (100 mg/m^2^ body surface area in weeks 1, 4, and 7) were administered concomitantly during radiotherapy.

### Imaging

All patients received computed tomography (CT), fluorine-18-deoxyglucose ([^18^F]FDG), and [^18^F]FMISO PET/CT imaging at baseline, while [^18^F]FMISO PET/CT scans were repeated in weeks 2 and 5 during chemoradiation as described earlier [33]. PET/CT imaging was performed on a Gemini TrueFlight PET/CT scanner (Philips, Hamburg, Germany). A total of 3.7 MBq/kg [^18^F]FMISO was administered intravenously to a maximum activity of 370 MBq, and PET/CT imaging was performed in radiation treatment position using a thermoplastic head immobilization mask at 150 min postinjection. This time interval was chosen to assure that [^18^F]FMISO can adequately diffuse from the microvessels to very distant tumor microareas (up to a distance of approx. 950 μm, calculated using Einstein’s equation for one-dimensional diffusion), to improve the detection of acute hypoxia in the clinical setting and to improve signal-to-noise-ratio.

PET/CT images were co-registered with the corresponding planning CTs. As described previously, GTVs both for the primary tumor and metastatic lymph nodes were delineated on the [^18^F]FDG-PET/CT co-registered images (if available, MRI was also co-registered and used for target volume delineation) [34]. A region of interest (ROI) within the contralateral sternocleidomastoid muscle was delineated, and mean [^18^F]FMISO SUV was determined for this ROI. Voxels within the primary and nodal GTVs were considered as hypoxic if the ratio of [^18^F]FMISO SUV to mean SUV in the contralateral sternocleidomastoid muscle was in excess of 1.4. The volume of the tumoral hypoxic subvolumes (HSVs) within the primary and nodal GTVs as well as the maximum [^18^F]FMISO SUV tumor-to-muscle ratio (i.e., maximum [^18^F]FMISO SUV within the primary tumor/mean [^18^F]FMISO SUV within the ROI inside the contralateral sternocleidomastoid muscle) was calculated at baseline as well as in treatment weeks 2 and 5. Blood sampling was performed at the times of the corresponding [^18^F]FMISO PET/CT scans.

### Blood sampling and analyses

Patient blood was collected in EDTA monovettes® (Sarstedt, Nümbrecht, Germany), and collection tubes were cooled on ice and centrifuged at 500g for 10 minutes at 4 °C. Afterwards, serum supernatant was aliquoted into Nalgene™ Cryogenic storage tubes (Nalgene® Labware, Rochester, NY, USA) and stored at −80 °C. Enzyme-linked immunosorbent assays (ELISAs) for IL-6 (Human IL-6 Quantikine ELISA Kit, D6050, R&D Systems, Minneapolis, MN, USA) were used following the manufacturers’ instructions. The technician who performed the ELISA analyses was blinded regarding both tumor hypoxia data and patient outcomes.

### Statistical analyses

Parametric variables such as IL-6 concentration, [^18^F]FMISO T/M-ratio, and HSV were given as mean values with standard deviations. IL-6 and HSV dynamics during treatment were examined using mixed model analyses with post-hoc Tukey tests. Differences regarding patients’ IL-6 levels in dependence of clinical and pathological parameters were investigated with unpaired *t* tests (2 groups) or ANOVA tests (≥3 groups). Pearson’s correlations were carried out to determine correlations between HSVs and IL-6 plasma concentration. Clinical and tumor-related parameters that could be associated with baseline IL-6 levels (BMI, ECOG, smoking, T stage, N stage, HPV status, tumor volume, [^18^F]FMISO T/M-ratio, HSV) were included in the multiple linear regression model in which all variables were entered into the equation in one step (enter method). Receiver-operating characteristic (ROC) analyses were performed to determine sensitivity and specificity of different IL-6 cutoffs regarding the prediction of Δ[^18^F]FMISO T/M-ratio dynamics between weeks 0 and 2. Cox regression analyses were performed for IL-6 concentrations (used as continuous variables) in terms of locoregional control (LRC), progression-free survival (PFS), and overall survival (OS), and hazard ratios (HR) with the corresponding 95% confidence intervals (95% CI) were presented. Outcomes were also quantified using Kaplan-Meier analyses with log-rank tests. *p*≤0.05 was considered statistically significant. SPSS Statistics software version 25 (IBM, Armonk, NY, USA) and GraphPad version 8.2.1 (GraphPad Software, San Diego, CA, USA) were used for statistical analyses.

## Results

### IL-6 concentration increases over the course of chemoradiation

**Fig. 1:**
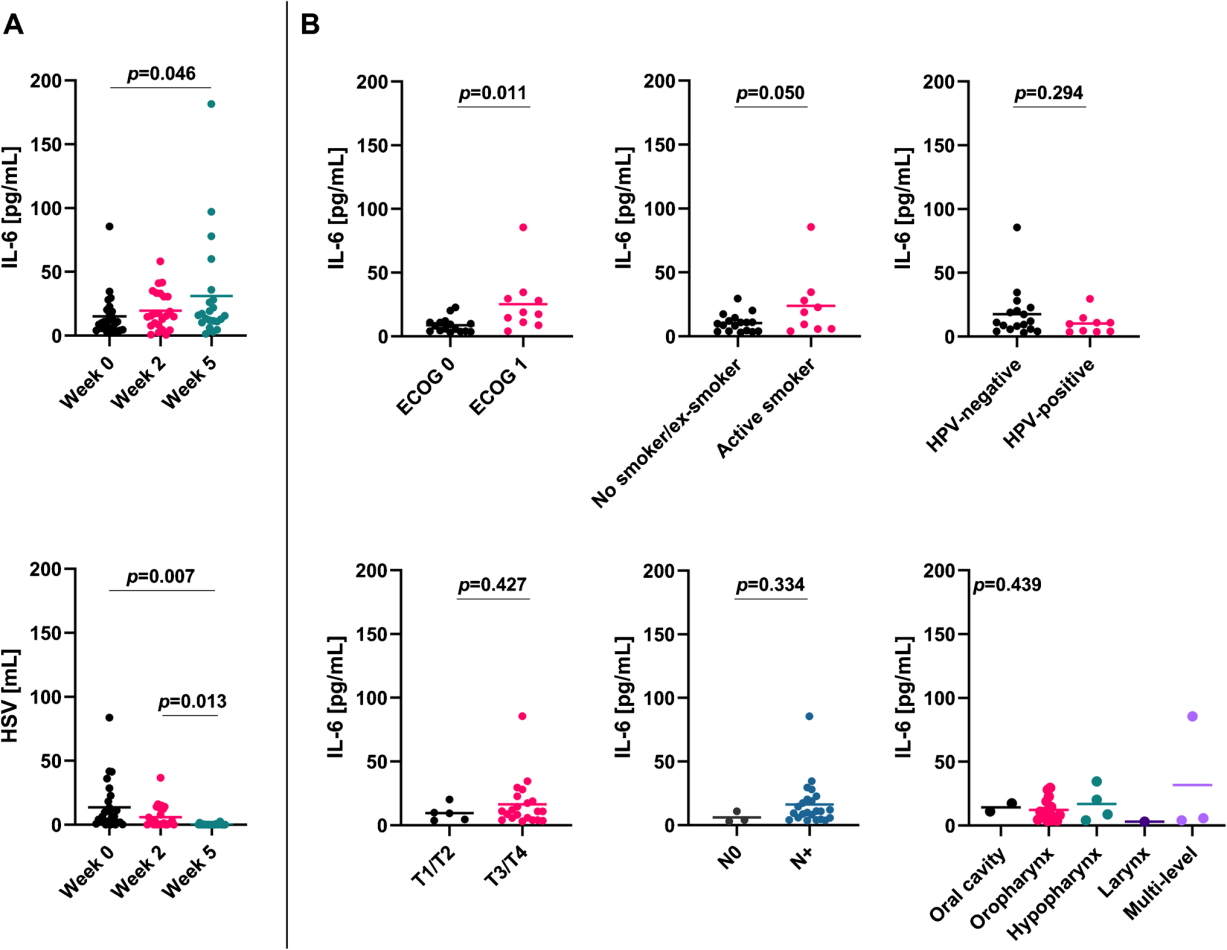
Smoking and reduced performance status go along with higher pre-therapeutic IL-6 levels. **A** IL-6 concentration and total tumoral HSV over the course of chemoradiation. Groups were compared using a mixed model analysis with post-hoc Tukey tests. **B** Baseline IL-6 plasma concentration in dependence of patients’ performance status, smoking status, HPV status, T stage, N stage, and tumor localization. Groups were compared with unpaired *t* tests (except for localization, in which an ANOVA test was applied). ECOG, Eastern Cooperative Oncology Group; HPV, human papillomavirus; HSV, hypoxic subvolume; IL-6, interleukin 6

Mean IL-6 plasma concentration ranged at 15.1 ±16.8 pg/mL, 19.6 ±15.1 pg/mL, and 31.0 ±41.5 pg/mL in weeks 0, 2, and 5, respectively (Fig. 1). As repeated measures ANOVA cannot handle missing data which was the case in few patient (*n*=3 in week 2, *n*=5 in week 5), we instead analyzed the data by fitting a mixed model. Here, we observed a significant increase of IL-6 plasma levels between weeks 0 and 5 of chemoradiation (*p*=0.037 for the mixed model, *p*=0.046 for the comparison between week 0 and 5). The mean difference of patient IL-6 levels was 5.3 ±17.2 pg/mL between weeks 0 and 2 and 17.6 ±29.6 pg/mL between weeks 0 and 5. Eight of 23 patients (34.8%) exhibited a decrease in IL-6 within the first two weeks, and 4 of 22 patients (18.2%) showed lower IL-6 levels in week 5 compared to week 0.

Total tumoral HSV (HSV within the primary GTV plus HSV within nodal GTVs) amounted to 13.6 ±18.9 mL, 6.0 ±8.6 mL, and 0.3 ±0.6 mL in weeks 0, 2, and 5, respectively. Mixed model analyses revealed a significant decrease of HSVs between week 0 and 5 (*p*=0.007) as well as between week 2 and 5 (*p*=0.013).

### IL-6 levels correlate with clinical characteristics

A reduced ECOG performance status was associated with considerably higher baseline IL-6 plasma concentrations (25.3 versus 8.7 pg/mL, *p*=0.011, unpaired *t* test). Patients who were smokers at the time of chemoradiation had significantly higher pre-therapeutic IL-6 levels than nonsmokers/ex-smokers (*p*=0.050). In our cohort, there was no difference between patients with HPV-positive and HPV-negative tumors regarding IL-6 concentrations prior to chemoradiation (*p*=0.294). Furthermore, we could not detect differences depending on T stages (*p*=0.427), N stages (*p*=0.334), or tumor localization (*p*=0.439, ANOVA test). There was an inverse correlation between baseline BMI and IL-6 plasma levels (Pearson’s r=−0.400, *p*=0.043).

### HSV correlates with IL-6 plasma levels

**Fig. 2:**
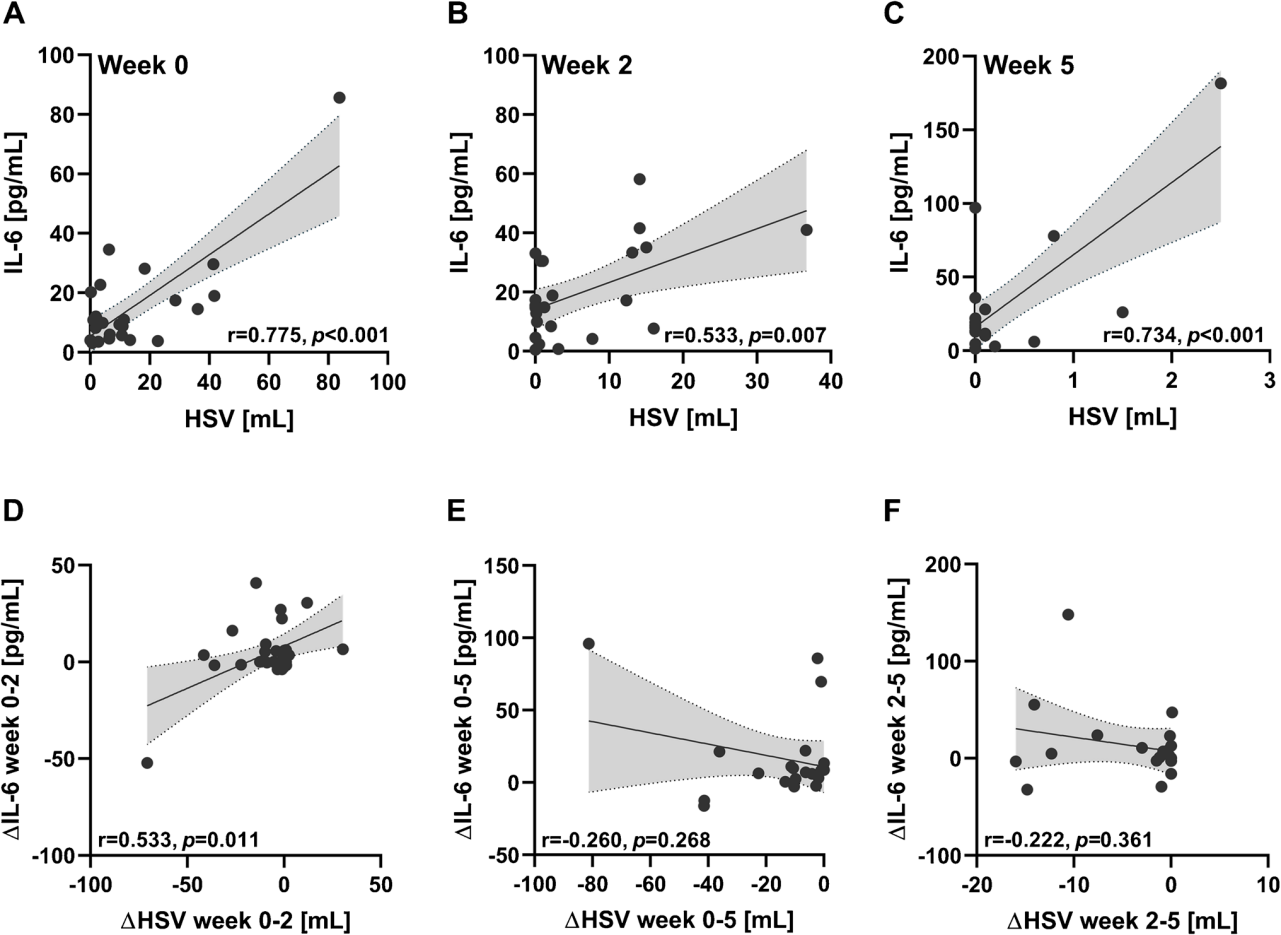
IL-6 correlates with tumor’s hypoxia volume. **A–C** Correlative analyses between HSV and IL-6 plasma levels in week 0 (**A**), week 2 (**B**), and week 5 (**C**). **D–F** HSV dynamics between week 0 and 2 (**D**), between week 0 and 5 (**E**), and between week 2 and 5 (**F**) were correlated with the associated IL-6 plasma levels dynamics. Pearson’s *r* values with the according *p* values as well as regression lines with the corresponding 95% CI are presented

We performed Pearson’s correlation analyses between total tumoral HSVs and IL-6 plasma concentrations. Both parameters showed a positive correlation with each other in treatment week 0 (*r*=0.775, *p*<0.001), week 2 (*r*=0.533, *p*=0.007), and week 5 (*r*=0.734, *p*<0.001) (Fig. 2). Two representative patient cases are shown in Supplementary Fig. 1. HSV dynamics within the first 2 weeks of chemoradiation were found to correlate with IL-6 dynamics (*r*=0.533, *p*=0.011), whereas there was no correlation in the other time intervals (week 0–5: *r*=−0.260, *p*=0.268, week 2–5: *r*=−0.222, *p*=0.361). However, when the outlier (Fig. 2D) was removed, the correlation between ΔHSV_week 0-2_ and ΔIL-6_week0-2_ was no longer detectable (*r*=0.094, *p*=0.687). In contrast to the significant associations between patient HSVs and IL-6 plasma levels, there was no significant correlation between [^18^F]FMISO T/M-ratio and IL-6 plasma concentration in week 0 (*r*=0.283, *p*=0.253), week 2 (*r*=0.370, *p*=0.083), and week 5 (*r*=0.275, *p*=0.240) (Supplementary Fig. 2).

We examined whether HSV remains a significant predictor for IL-6 plasma concentration if other patient- and tumor-related variables potentially influencing IL-6 levels were included into a multiple regression analysis (Table 2). Here, baseline HSV was the only parameter (*p*=0.045) that significantly predicted IL-6 levels, whereas BMI (*p*=0.334), ECOG (*p*=0.448), smoking (*p*=0.728), T stage (*p*=0.583), N stage (*p*=0.880), HPV status (*p*=0.708), tumor volume (*p*=0.137), and [^18^F]FMISO T/M ratio (*p*=0.651) had no significant impact.

**Table 2:** Multiple regression of several parameters regarding IL-6 prediction at baseline. BMI, body mass index; ECOG, Eastern Cooperative Oncology Group; HPV, human papillomavirus; HSV, hypoxic subvolume; IL-6, interleukin-6; RT, radiotherapy; T/M-ratio, tumor-to-muscle ratio; wk, week

**Variable**	**Unstandardized B**	***p***
BMI (continuous)	−0.533	0.334
ECOG	5.231	0.448
Smoking during RT	−1.154	0.728
T stage	−3.761	0.583
N stage	1.163	0.880
HPV status	−2.263	0.708
Tumor volume wk0 (continuous)	0.086	0.137
[^18^F]FMISO T/M-ratio (continuous)	−1.873	0.651
HSV wk0 (continuous)	0.443	**0.045**

### Lack of an early hypoxia response correlates with increased IL-6 levels in treatment week 2

**Fig. 3:**
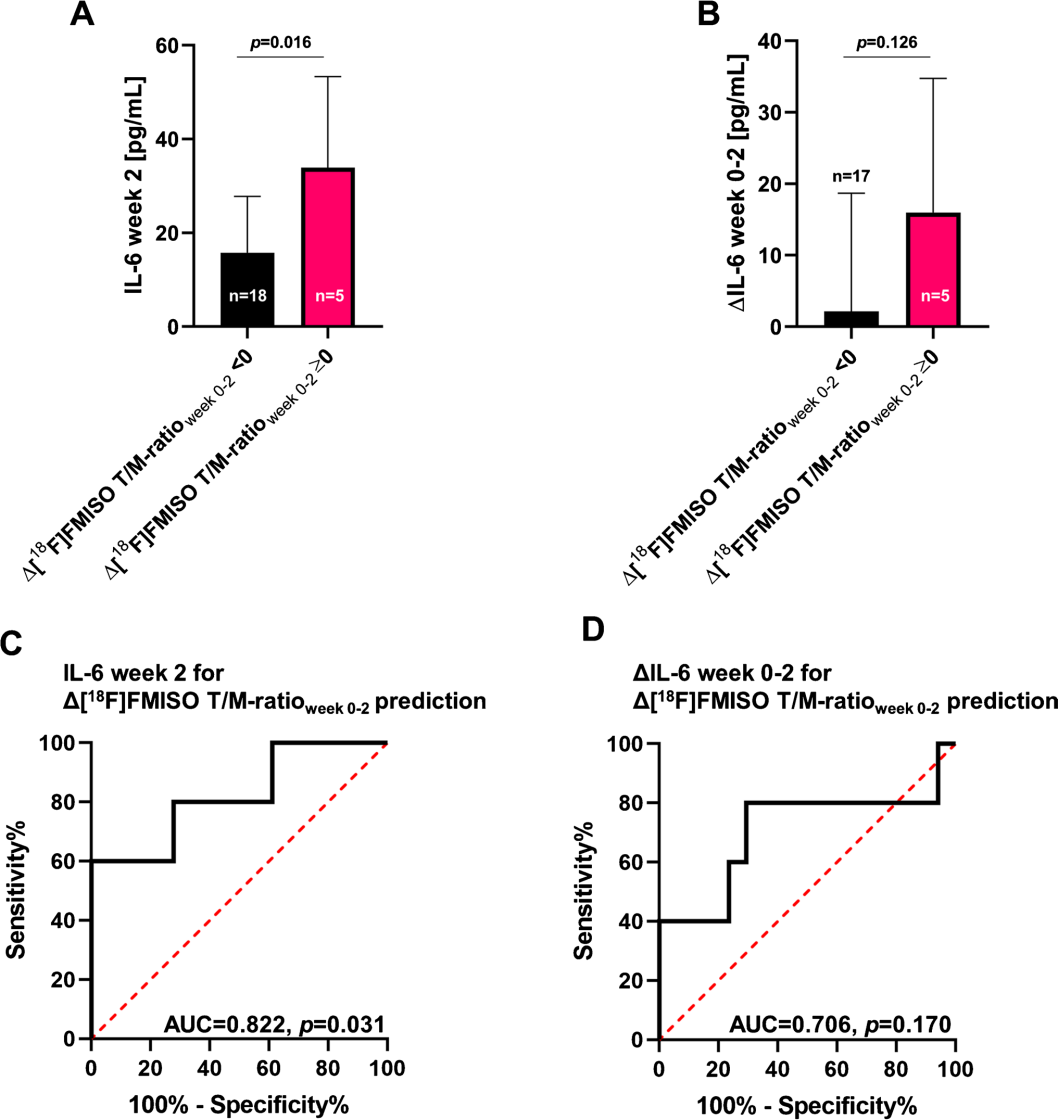
IL-6 concentration in week 2 predicts Δ[^18^F]FMISO T/M-ratio_week 0-2_. **A–B** Mean IL-6 plasma levels (**A**) and IL-6 dynamics within the first 2 weeks (**B**) depending on Δ[^18^F]FMISO T/M-ratio_week 0-2_. Data are shown as mean ± standard deviation, and groups were compared with unpaired *t* tests. [^18^F]FMISO T/M-ratio is defined as maximum [^18^F]FMISO SUV within the tumor divided by the mean SUV within the contralateral sternocleidomastoid muscle. **C–D** ROC analyses of IL-6 plasma values in week 2 (**C**) and IL-6 dynamics from baseline to week 2 (**D**) in terms of Δ[^18^F]FMISO T/M-ratio_week 0-2_ prediction. AUC values with corresponding *p* values are given

Due to the significant association between HSV and IL-6 dynamics within the first 2 weeks of chemoradiation, we tested whether IL-6 plasma levels in week 2 varied based on an early PET-based hypoxia response, defined as Δ[^18^F]FMISO T/M-ratio_week 0-2_ <0 (Fig. 3). In previous studies, we and others could show that an early hypoxia response is a key prognostic parameter for the outcome of HNSCC patients undergoing chemoradiation [32, 34]. Patients with absent tumor hypoxia response within the first 2 weeks of treatment (Δ[^18^F]FMISO T/M-ratio_week 0-2_ ≥0) exhibited elevated mean IL-6 plasma levels that were more than twice as high as in patients with Δ[^18^F]FMISO T/M-ratio_week 0-2_ <0 (33.9 versus 15.8 pg/mL, *p*=0.016). Furthermore, IL-6 dynamics between weeks 0 and 2 were found to differ depending on patients’ early PET-based hypoxia response: While patients with absent tumor hypoxia response within the first 2 weeks of treatment exhibited a considerable increase of IL-6 plasma levels (Δ16.0 ng/mL), patients with early hypoxia response (Δ[^18^F]FMISO T/M-ratio_week 0-2_ <0) were found to have rather stable IL-6 levels (Δ2.1 pg/mL, *p*=0.126).

### Plasma IL-6 concentration is able to predict early tumor hypoxia response during chemoradiation

ROC analyses were conducted to determine the potential of IL-6 levels in week 2 and ΔIL-6_week 0-2_ for predicting Δ[^18^F]FMISO T/M-ratio_week 0-2_. Using a cutoff value of 18.1 pg/mL for IL-6 in week 2, IL-6 concentration at this time point was able to predict tumor hypoxia response with a sensitivity of 80.0% and a specificity of 72.2%. The Youden index of this model was 0.52, and the AUC of the ROC analysis amounted to 0.822 (*p*=0.031). Differences in the IL-6 levels between baseline and week 2 had a reduced predictive value for Δ[^18^F]FMISO T/M-ratio_week 0-2_: AUC amounted to 0.706 (*p*=0.170), and by using Δ5.5 pg/mL as discrimination value (value chosen by maximizing Youden index approach), sensitivity and specificity were 80.0% and 64.7%, respectively.

### IL-6 has prognostic significance in HNSCC patients undergoing chemoradiation

A potential prognostic value of IL-6 in terms of LRC, PFS, and OS was investigated using Cox regression analyses (Table 3). Baseline IL-6 levels were found to result in a trend towards reduced LRC (HR=1.023, 95% CI 0.994–1.053, *p*=0.116), PFS (HR=1.030, 95% CI 0.996–1.065, *p*=0.081), and OS (HR=1.027, 95% CI 0.996–1.058, *p*=0.090). IL-6 plasma levels in week 2 were not associated with LRC (HR=0.979, 95% CI 0.926–1.034, *p*=0.444), PFS (HR=1.008, 95% CI 0.971–1.047, *p*=0.663), and OS (HR=1.037, 95% CI 0.994–1.082, *p*=0.093) in our dataset. However, IL-6 levels in week 5 were observed to correspond to significantly deteriorated OS (HR=1.018, 95% CI 1.004–1.033, *p*=0.013) and a trend towards reduced PFS (HR=1.013, 95% CI 0.999–1.013, *p*=0.078), whereas LRC was not significantly influenced (HR=1.008, 95% CI 0.993–1.023, *p*=0.324). The Kaplan-Meier OS curves for patients with high or low IL-6 levels at week 5 are shown as Supplementary Fig. 3.

**Table 3:** Cox proportional hazards regression analyses for IL-6 plasma levels regarding LRC, PFS, and OS. CI, confidence interval; HR, hazard ratio; IL-6, interleukin-6; LRC, locoregional control; OS, overall survival; PFS, progression-free survival; wk, week

	LRC			PFS			OS		
	HR	95% CI	*p*	HR	95% CI	*p*	HR	95% CI	*p*
IL-6 wk0	1.023	0.994–1.053	0.116	1.030	0.996–1.065	0.081	1.027	0.996–1.058	0.090
IL-6 wk2	0.979	0.926–1.034	0.444	1.008	0.971–1.047	0.663	1.037	0.994–1.082	0.093
IL-6 wk5	1.008	0.993–1.023	0.324	1.013	0.999–1.013	0.078	1.018	1.004–1.033	**0.013**

## Discussion

Based on the present data from a prospective exploratory imaging trial, we could demonstrate a strong association between IL-6 plasma levels and PET imaging-derived tumor hypoxia dynamics in patients undergoing chemoradiation for locally advanced HNSCC. Importantly, the magnitude of tumor hypoxia (i.e., HSV) remained the only significant parameter for IL-6 plasma concentrations in the multiple regression analysis. Furthermore, IL-6 dynamics within the first two treatment weeks were found to positively correlate with the prognostically relevant early hypoxia dynamics in [^18^F]FMISO-PET/CT [17, 32, 35, 36].

IL-6 was discovered in 1986 as a B cell stimulatory factor; it is a multifunctional and pleiotropic cytokine that is secreted by several cell types such as monocytes, lymphocytes, keratinocytes, and endothelial and tumor cells [37, 38]. IL-6 has been established as an important regulator of the immune system and modulates different biological processes, e.g., inflammation, immunity, hematopoiesis, cellular proliferation and differentiation, angiogenesis, apoptosis, and carcinogenesis [38]. Hypoxia has been shown to increase IL-6 secretion in vitro in several cell types [26, 39, 40], and hypoxia-induced NF-κB and NF-IL-6 activation was found to be partly responsible for hypoxia-induced transcriptional activation of the IL-6 gene [39, 41]. Additionally, platelet-activating factor and platelet-derived growth factor have been shown to mediate hypoxia-induced IL-6 production [26]. Given these preclinical observations, our findings regarding the correlation between IL-6 levels and [^18^F]FMISO-PET imaging-assessed tumor hypoxia seem biologically plausible.

IL-6 has been reported as a prognostic parameter for several tumors including HNSCC [22, 23]. Increased radio- and chemoresistance, higher proliferation, elevated invasion, and metastatic potential as well as promoted epithelial-mesenchymal transition of hypoxic HNSCC could all contribute to the prognostic value of IL-6. Our observations suggest that hypoxia-linked IL-6 expression is a further potential mechanism by which IL-6 worsens the prognosis of HNSCC patients. IL-6 is known to increase resistance to ionizing radiation and cisplatin that may explain why there was a positive association between IL-6 dynamics and early treatment responses regarding tumor-associated hypoxia [42, 43]. Both hypoxia and IL-6 are able to transform non-stem cancer cells into cancer stem cells, and the intrinsic radioresistance of tumor stem cells may further explain the observed association between tumor hypoxia, IL-6 levels, and patient outcomes [44–47].

We found a significant increase of IL-6 during chemoradiation that has already been described by other groups [48, 49]. Both ionizing radiation and cisplatin have been shown to upregulate IL-6 expression in HNSCC in vitro and in vivo [50]. We therefore hypothesize that the significant increase of IL-6 during chemoradiation is a multifactorial process including radiotherapy and concurrent cisplatin administration, mucositis, and malnutrition. Chemoradiation-induced hypoxia resolution at week 5, as measured by [^18^F]FMISO-PET/CT, occurred in the vast majority of patients, and may have weakened the increase of IL-6 plasma levels during treatment. The multifactorial process underlying the observed IL-6 increase during chemoradiation could explain why we observed no correlation between IL-6 and HSV dynamics towards the end of treatment, i.e., between weeks 2 and 5.

Despite presenting longitudinal imaging and biological data from a prospective trial and providing a potential link between IL-6 and tumor hypoxia in HNSCC patients treated by chemoradiation, there are some limitations, especially the limited sample size and absent external validation. Rübe et al. have shown for non-small cell lung cancer (NSCLC) that the tumor itself is the major source of plasma IL-6; however, we cannot conclusively prove that the measured plasma IL-6 only derive from tumor cells in this trial [51].

Upon external validation, our data may be of clinical relevance for personalized radiotherapy in the future: Considering the prognostic value of IL-6 concentrations in week 5, IL-6 could be used as peritherapeutic biomarker in order to select patients with an unfavorable prognosis suitable for treatment escalation, e.g., using a sequential boost concept. On the other hand, given the considerable treatment-related toxicities of chemoradiation for HNSCC patients, many treatment de-escalation approaches (mainly for HPV-positive oropharyngeal cancer patients) are currently investigated. Absent baseline hypoxia and early hypoxia resolution have been suggested to be relevant parameters to select appropriate patients for de-escalation approaches, and initial pilot studies applying treatment de-escalation have shown promising results [16, 17]. As the logistic efforts of repeat [^18^F]FMISO-PET/CT during chemoradiation are considerable, surrogate parameters for dynamic hypoxia PET imaging are desirable, and blood- and tissue-based biomarkers, gene signatures, and multiparametric MRI have been proposed to serve as surrogate markers to detect and monitor tumor hypoxia [11, 52–57]. However, carbonic anhydrase IX, osteopontin, and vascular endothelial growth factor plasma levels did not correlate with [^18^F]HX4-PET-measured hypoxia parameters in a previous small study [58], showing the difficulty to find appropriate blood hypoxia markers.

We and other groups have previously shown that early hypoxia dynamics during treatment rather than baseline hypoxia provides prognostic relevance in HNSCC patients; therefore, there is strong need for endogenous hypoxia markers that can easily be studied longitudinally during chemoradiation [32, 35, 36, 59]. In this respect, IL-6 may serve as an easily obtainable surrogate hypoxia parameter that could be beneficial for patient stratification required for treatment personalization approaches.

In summary, IL-6 plasma levels strongly correlated with [^18^F]FMISO PET-detected intratumoral hypoxia in HNSCC patients and were able to significantly predict a prognostically favorable early hypoxia response within the first 2 weeks of chemoradiation. Our data suggest that IL-6 may serve as an easily obtainable endogenous blood hypoxia marker that may allow patient stratification for hypoxia-directed personalized radiotherapy concepts.

## Supplementary Information

ESM 1 (PNG 19783 kb)

High resolution image (TIF 1203 kb)

ESM 2 (PNG 19783 kb)

High resolution image (TIF 716 kb)

ESM 3 (PNG 19783 kb)

High resolution image (TIF 574 kb)

## Data Availability

Research data are stored in an institutional repository and will be shared upon reasonable request to the corresponding author.

## Code availability

Non applicable.
