# Supplementary figures and images for: Interleukin-6 as surrogate marker for imaging-based hypoxia dynamics in patients with head-and-neck cancers undergoing definitive chemoradiation—results from a prospective pilot trial
Source: Eur J Nucl Med Mol Imaging. 2021 Nov 13;49(5):1650–60. doi: 10.1007/s00259-021-05602-x (PMC8940848; doi:10.1007/s00259-021-05602-x)

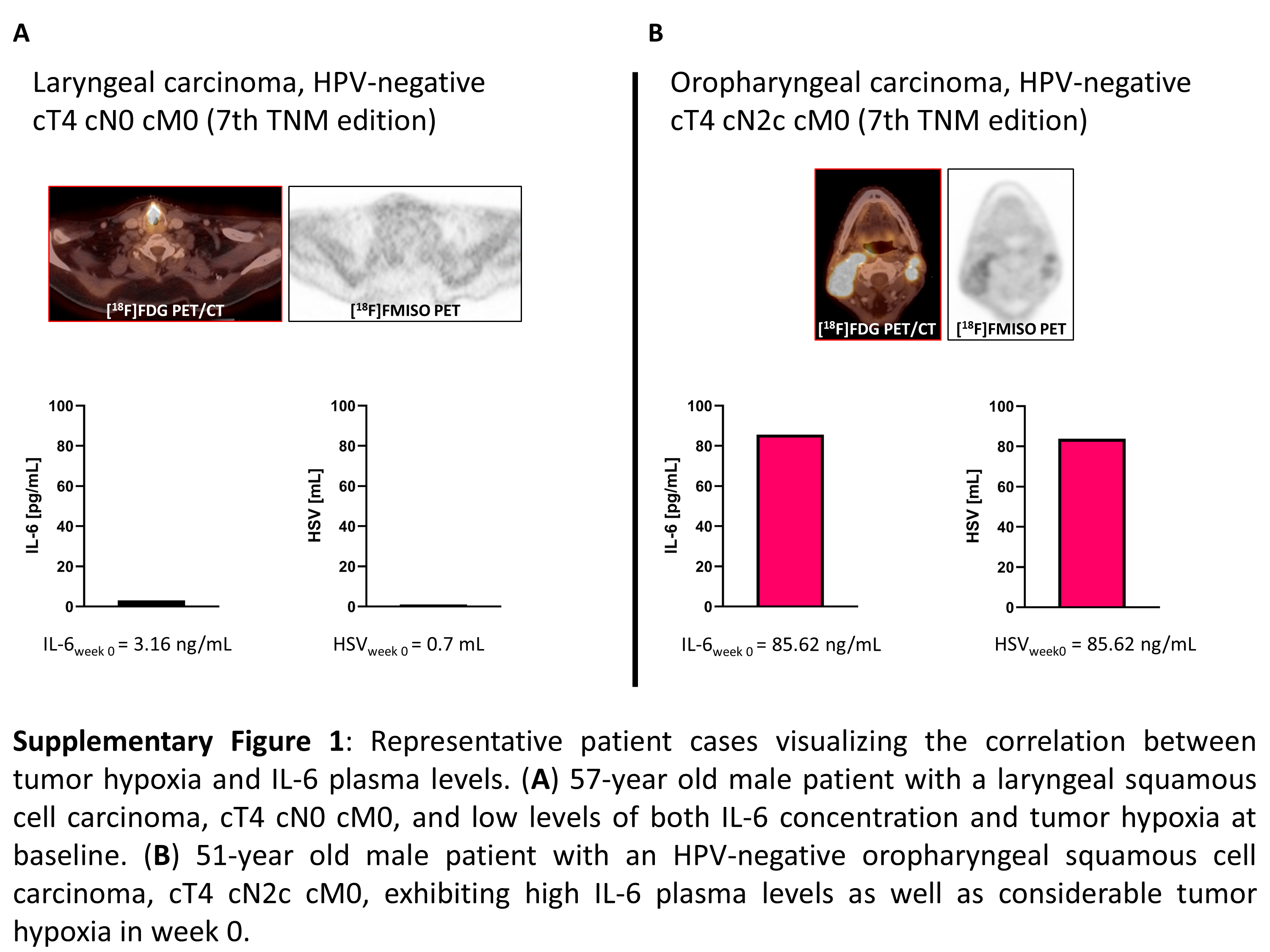

Supplement: Supplementary file 1 — (PNG 19783 kb) [file 259_2021_5602_Fig4_ESM.png]

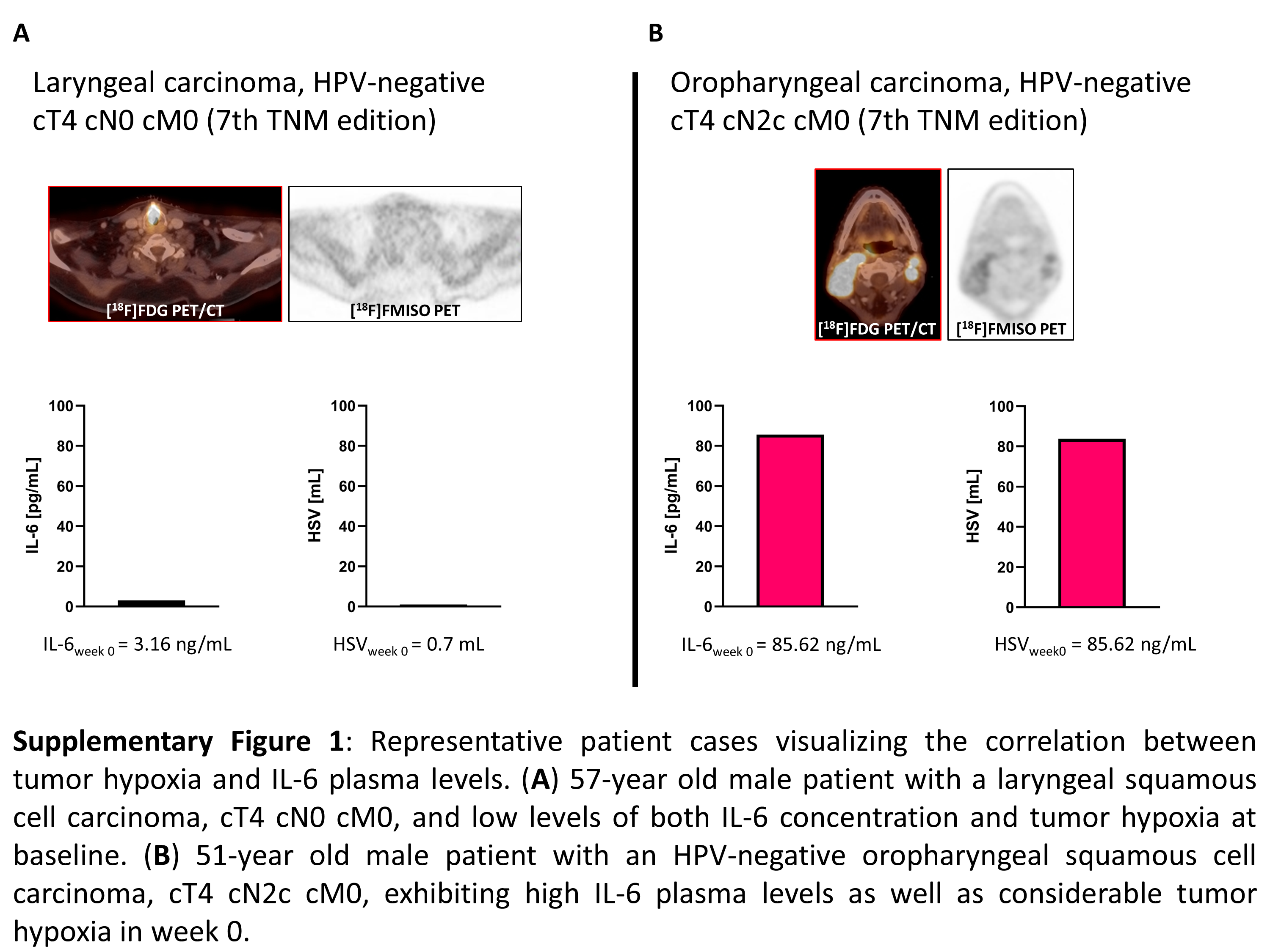

Supplement: Supplementary file 2 — High resolution image (TIF 1203 kb) [file 259_2021_5602_MOESM1_ESM.tif]

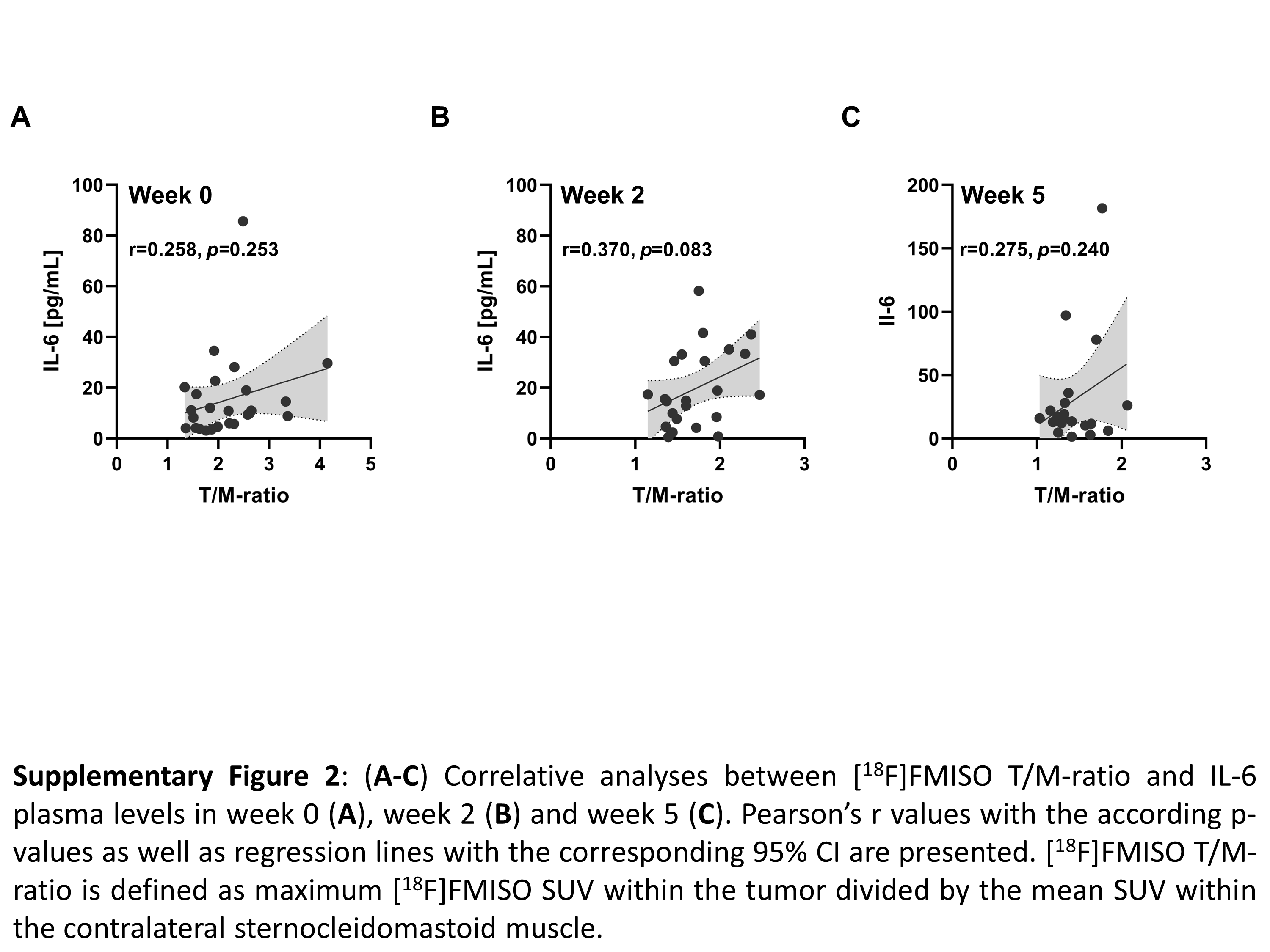

Supplement: Supplementary file 3 — (PNG 19783 kb) [file 259_2021_5602_Fig5_ESM.png]

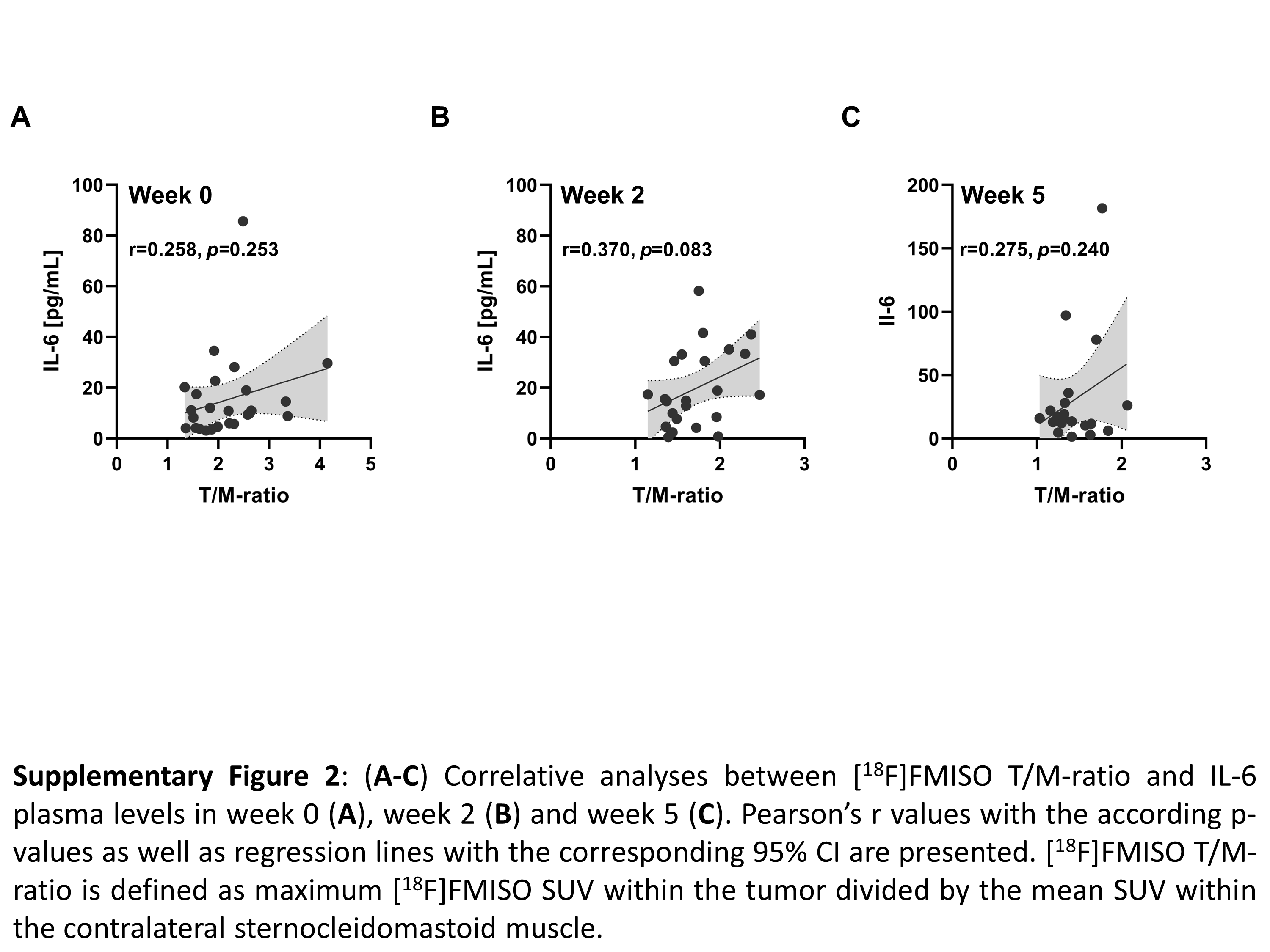

Supplement: Supplementary file 4 — High resolution image (TIF 716 kb) [file 259_2021_5602_MOESM2_ESM.tif]

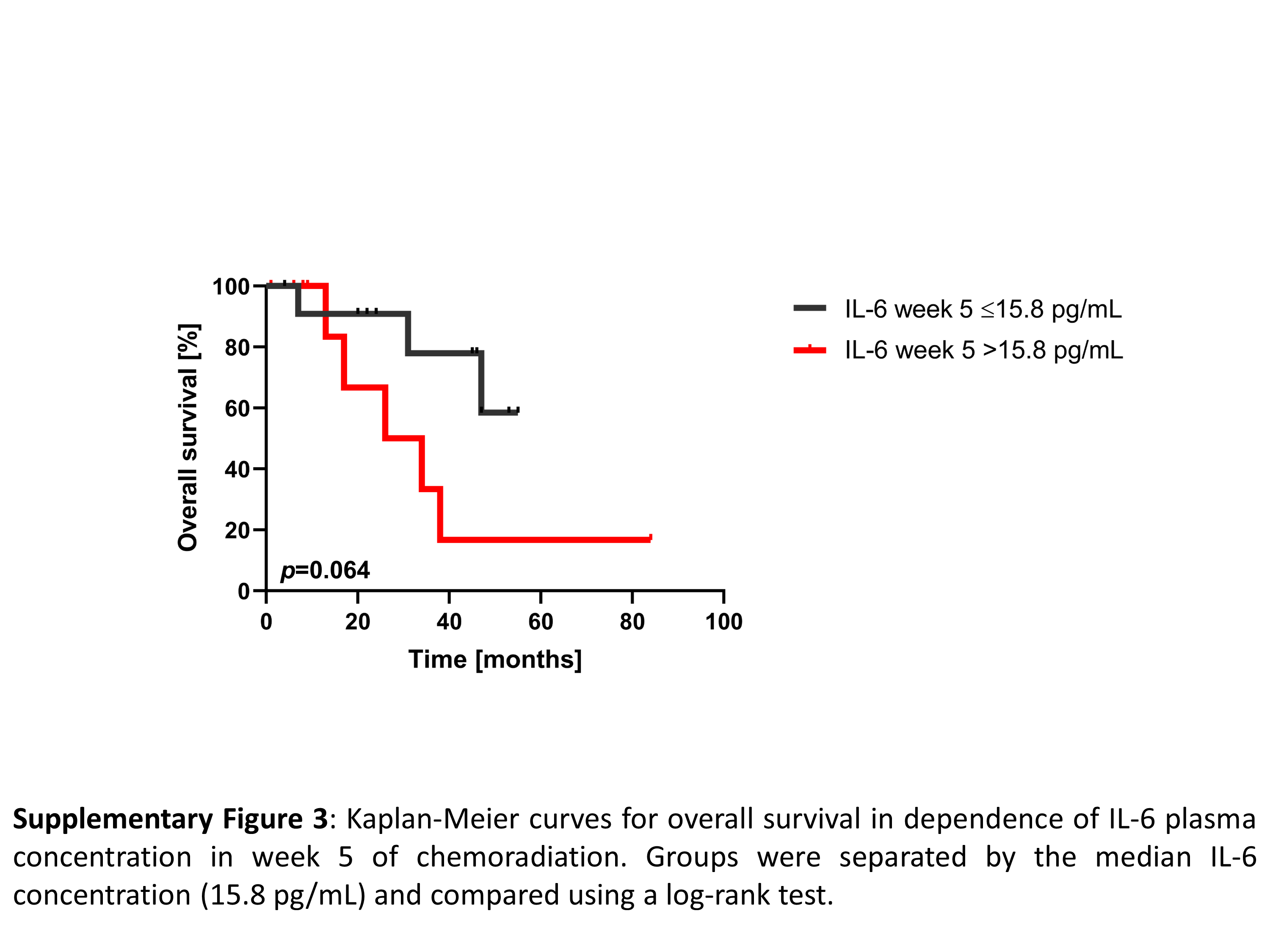

Supplement: Supplementary file 5 — (PNG 19783 kb) [file 259_2021_5602_Fig6_ESM.png]

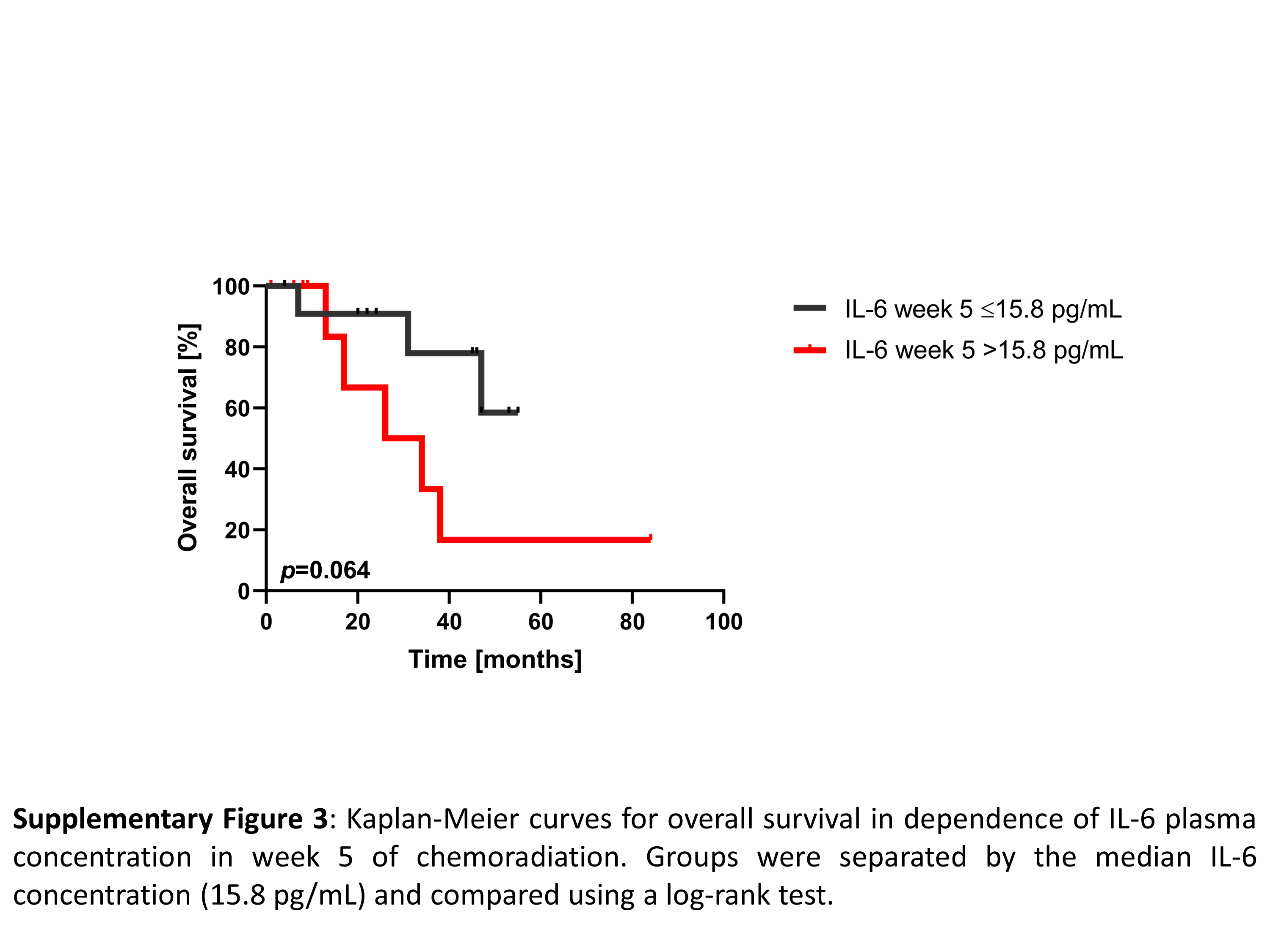

Supplement: Supplementary file 6 — High resolution image (TIF 574 kb) [file 259_2021_5602_MOESM3_ESM.tif]
